# Photomutagenicity of chlorpromazine and its *N*-demethylated metabolites assessed by NGS

**DOI:** 10.1038/s41598-020-63651-y

**Published:** 2020-04-23

**Authors:** José A. G. Agúndez, Elena García-Martín, Guillermo García-Lainez, Miguel A. Miranda, Inmaculada Andreu

**Affiliations:** 1 University Institute of Molecular Pathology Biomarkers, UEx. ARADyAL, Instituto de Salud Carlos III, 10003 Cáceres, Spain; 2 Instituto de Investigación Sanitaria (IIS) La Fe, Hospital Universitari i Politècnic La Fe, Avenida de Fernando Abril Martorell 106, 46026 Valencia, Spain; 3 Departamento de Química-Instituto de Tecnología Química UPV-CSIC. Universitat Politècnica de València, Camino de Vera s/n, Apdo 22012, 46071 Valencia, Spain; 4 Unidad Mixta de Investigación UPV-Instituto de Investigación Sanitaria (IIS) La Fe, Hospital Universitari i Politècnic La Fe, Avenida de Fernando Abril Martorell 106, 46026 Valencia, Spain

**Keywords:** Chemical biology, DNA

## Abstract

The human genome is constantly attacked by endogenous and exogenous agents (ultraviolet light, xenobiotics, reactive oxygen species), which can induce chemical transformations leading to DNA lesions. To combat DNA damage, cells have developed several repair mechanisms; however, if the repair is defective, DNA lesions lead to permanent mutations. Single-cell gel electrophoresis (COMET assay) is a sensitive and well-established technique for quantifying DNA damage in individual cells. Nevertheless, this tool lacks relationship with mutagenesis. Therefore, to identify errors that give rise to mutations it would be convenient to test an alternative known procedure, such as next generation sequencing (NGS). Thus, the present work aims to evaluate the photomutagenicity of neuroleptic drug chlorpromazine (CPZ), and its *N*-demethylated metabolites using COMET assay and to test NGS as an alternative method to assess photomutagenesis. In this context, upon exposure to UVA radiation, COMET assay reveals CPZ-photosensitized DNA damage partially repaired by cells. Conversely with this result, metabolites demethylchlorpromazine (DMCPZ) and didemethylchlorpromazine (DDMCPZ) promote extensive DNA-photodamage, hardly repaired under the same conditions. Parallel assessment of mutagenesis by NGS is consistent with these results with minor discrepancies for DDMCPZ. To our knowledge, this is the first example demonstrating the utility of NGS for evaluating drug-induced photomutagenicity.

## Introduction

The human genome is exposed to continuous attack by endogenous and exogenous genotoxic stresses (ultraviolet light, ionizing radiation, xenobiotics, reactive oxygen species, etc). These agents can induce chemical transformations leading to the most common DNA lesions: base and sugar modifications, alkylation, single strand breaks (ssb) generated by oxidation, and spontaneous hydrolysis. In this context, cells have developed several DNA-repair pathways; however, if damaged DNA is not properly repaired, DNA lesions may lead to permanent mutations in the genetic code as a consequence of the change in the sequence of base pairs^1–7^. For detecting genotoxicity in single cells, a very versatile, sensitive and well-established technique is the single-cell gel electrophoresis or COMET assay^8–11^. This tool is based on an electrophoresis with DNA of lysed, agarose-embedded cells, also known as nucleoids, which upon fluorescent staining appear a comet-like image. Thus, the comet head is composed of intact DNA, while the tail consists of single-strand or double-strand DNA breaks. The relative amount of DNA migrating away from the head of the comet is directly proportional to the extent of DNA damage. However, this data indicates DNA alterations but it does not necessarily relate to mutagenesis. Thus, the lack of a close relationship between DNA migration in the COMET assay and mutagenesis may be explained because the former detects unrepaired DNA, while in the latter only the misrepaired errors are considered. Therefore, in order to identify errors that might give rise to mutations it would be convenient to test the potential of an alternative known platform such as next generation sequencing (NGS) or high-throughput sequencing^12–15^. These techniques allow an accurate assessment of mutations provided that the gene area covered is large, and the sequencing is deep enough, and they are straightforward, can be easily standardized and, nowadays, are affordable.

**Figure 1:**
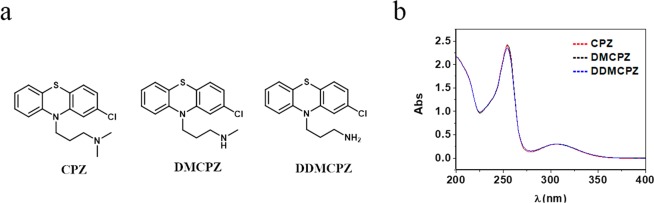
(**a**) Chemical structures of chlorpromazine (CPZ) and its *N*-demethylated metabolites demethylchlorpromazine (DMCPZ) and didemethylchlorpromazine (DDMCPZ). (**b**) UV absorption spectra of CPZ, DMCPZ, DDMCPZ in PBS at 10^−5^ M.

It is well known that chlorpromazine (CPZ), a neuroleptic drug used in the treatment of schizophrenia, can induce photosensitivity reactions^16,17^. Moreover, during phase I of metabolism, CPZ undergoes mono-*N*-demethylation and di-*N*-demethylation in the side chain. In this context, we have previously reported that demethylation of CPZ leads to metabolites demethylchlorpromazine (DMCPZ) and didemethylchlorpromazine (DDMCPZ), which maintain identical chromophore to the parent drug (Fig. 1), does not result in a detoxification but leads to an even enhanced photogenotoxicity^18^.

With this background, the aim of the present work is to evaluate the photomutagenicity of CPZ and its *N*-demethylated metabolites by means of the COMET assay and to test the potential of NGS as an alternative and straightforward procedure to assess photomutagenesis.

## Methods

### Chemicals and reagents

Chlorpromazine hydrochloride (CPZ) was provided by Sigma Aldrich (Madrid, Spain). Demethylchlorpromazine (DMCPZ) and didemethylchlorpromazine (DDMCPZ) were synthesized as previously reported^18^. Fetal bovine serum (FBS), Dulbecco’s Modified Eagle Medium (DMEM) and penicillin-streptomycin were purchased from Invitrogen (Madrid, Spain). Trypsin and L-glutamine solutions were supplied by Cultek (Madrid, Spain). Phosphate buffer saline sterile solutions (0.01 M, pH 7.4) were prepared in ultrapure water obtained from a Milli-Q water purification system. For single cell electrophoresis (COMET) assay, the electrophoretic tank and lysis solution were provided by Trevigen (Gaithersburg, USA), low melting point agarose was provided by Pronadisa (Madrid, Spain), and the SYBR Gold was obtained from Invitrogen. All other reagents were of HPLC grade and used without additional purification.

### Absorption spectral measurements

Ultraviolet absorption spectra of CPZ, DMCPZ and DDMCPZ were recorded on a Shimadzu UV-1800 UV/VIS spectrophotometer. Measurements were performed in PBS (10^−5^ M) using 1 cm quartz cells with 3.5 mL capacity at room temperature.

### Cell culture experiments

The human skin fibroblast cell line (FSK) was cultured in DMEM supplemented with 10% FBS, 4 mM L-glutamine and 1% penicillin/streptomycin. The cells were routinely maintained in exponential growth (weekly passages, 1:3 splitting ratio) in 75 cm^2^ plastic flasks in a humidified incubator at 37 °C under a 5% CO_2_ atmosphere. The day of the experiments, cells were first trypsinized, resuspended in cold PBS and allowed to recover at 4 °C for 2 hours (trypsin detachment generates mild DNA damage in FSK cell line). Then, FSK cells were seeded in duplicate at 1.0 × 10^5^ cell/well or 2.0 × 10^6^ cell/well in 24 or 6 well-plate for COMET assay or photomutagenicity studies respectively, and incubated at 37 °C in a CO_2_ incubator (100% relative humidity and dark conditions) with 10 μM of CPZ, DMCPZ or DDMCPZ solutions for 30 minutes. After incubation, one plate was placed in the multi-lamp photoreactor to irradiate the cells whereas the other one was kept in a dark box as a negative control.

Both COMET assay and photomutagenicity studies included ketoprofen (KP, 10 μM) as positive control. Cells were pretreated with KP at 37 °C in a CO_2_ incubator (100% relative humidity and dark conditions) for 30 minutes before the irradiation step.

### Irradiation procedure

All irradiation experiments were carried out with a photoreactor LZC-4 fitted with six top and eight side-UVA lamps (λ_max_ = 350 nm; Luzchem, Canada), which emit 94% UVA radiation and 2% UVB radiation. The plastic lid does not absorb beyond 310 nm, which contributes to the mitigation of the effect of UVB radiation over the cell cultures. The irradiance was determined using a calibrated power meter detector, resulting an irradiance of 7 mW/cm^2^.The irradiations were performed through the lid of the plates for five minutes in order to achieve a UVA dose equivalent to 2 J/cm^2^. The viability rate of treated cells after UVA irradiation was higher than 85%, indicating the suitability of the UV dose to overcome false-positive results triggered by extensive DNA fragmentation promoted by cell death. To prevent cell cultures from unwanted overheating, plates were kept on ice during the irradiation step and the temperature was regulated by ventilation.

### Evaluation of cellular photogenotoxicity by COMET assay

Irradiated and non-irradiated cell suspensions (100 μL) were carefully mixed with 100 μL of 1% low melting point agarose, and the drops were loaded on Trevigen treated slides placed on ice until jellification (2 × 10^4^ cells/gel). Then, slides were immediately subjected to cell lysis by incubation overnight in cold lysis buffer (2.5 M NaCl, 0.1 M Na_2_EDTA, 0.01 M Tris, 1% Triton X-100) or maintained in DMEM medium at 37 °C for 3 h and 6 h, and then lysed, to promote intrinsic cellular DNA-repair mechanisms. Next day, all slides were placed in the electrophoretic tank, covered with cold alkaline buffer (0.2 M NaOH, 1 mM EDTA, pH ≥13) and left during 40 min for DNA unwinding. Afterwards, the electrophoresis was carried out at 21 V (1 V/cm) for 30 minutes at 4 °C. Once the electrophoresis ended, the slides were washed twice in PBS for 5 min. DNA was fixed by two subsequent incubations in 70% ethanol and 100% ethanol. DNA comets and tails were stained with SYBR Gold (1:10.000) for 30 min (λ_exc_: 495 nm and λ_em:_ 537 nm). Finally, the slides were air-dried and kept in darkness until their visualization. Visualization of comet nucleoids and tails was carried out with a Leica DMI 4000B fluorescence microscope using the Fluorescein FITC filter. At least 5 pictures of each sample were taken in order to determine DNA damage. The percentage of DNA damage of each sample was calculated with the visual scoring of at least 100 DNA Comets using the following equation: [(Nclass 0 Comets × 0) + (Nclass 1 Comets × 1) + (Nclass 2 Comets × 2) + (Nclass 3 Comets × 3) + [(Nclass 4 Comets × 4) + (Nclass 5 Comets × 5) + (NClass 6 Comets × 6)]/6, where class 0 comets indicate comets with no DNA damage and class 6 comets indicate comets with maximum DNA damage.

### Photomutagenicity studies

Irradiated and non-irradiated samples were centrifuged at 1500 rpm for 5 min and cellular pellet was harvested and kept at −20 °C until DNA extraction. Thus, DNA extraction was carried out according to standard procedures, by using phenol/chloroform/isoamyl alcohol extraction and ethanol precipitation. The DNA was dissolved in sterile 100 mM Tris hydrochloric acid, pH 8.0, and 1 mM ethylendiaminetetraacetic acid at a final concentration of 400 to 600 μg/mL, and stored at 4 °C in sterile plastic vials. Mutagenicity studies were carried out by means of targeted deep sequencing (Ion PGM Targeted Sequencing, Life Technologies). *BRCA1* and *BRCA2* were selected as the reporter genes based on the availability of straightforward and inexpensive analyses and because of the high bp coverage comprised in the analyses. The chromosomal regions analysed correspond to well-known genes related to breast cancer since more than two decades ago^19,20^. The analyses covered 24.830 bp, 167 amplicons and the mean coverage was always higher than 340 x. Quality control data for the sequencing analyses are summarized in Table 1.

**Table 1:** Summary of the NGS quality control metrics in the samples studied.

Sample ID	Total number of reads	Total number of bases (mbp)	Mean coverage depth (fold)	Coverage within the target region (%)
CPZ_T0	71217	10.2638	448.05	100
CPZ_T5	79209	11.3726	496.37	100
FSK_T0	57750	8.3150	363.15	100
FSK_T5	55302	7.8122	341.58	100
DMCPZ_T0	83100	12.0291	525.74	99.82
DMCPZ_T5	87788	12.5787	549.36	99.98
DDMCPZ_T0	88685	12.7070	541.75	100
DDMCPZ_T5	86673	12.4188	542.69	100

## Results and Discussion

As stated above, in the photogenotoxicity studies for CPZ, DMCPZ and DDMCPZ carried out with cellular DNA, COMET assay revealed that the metabolites were more photogenotoxic than CPZ itself^18^. Therefore, to assess whether the cells can repair DNA damage photosensitized by CPZ and its *N*-demethylated metabolites after several hours, COMET assay of FSK cells treated with CPZ, DMCPZ or DDMCPZ was carried out after UVA light irradiation followed by 3 h or 6 h of cell recovery. Thus, the remaining DNA damage was calculated by using a classification of 6 DNA damage categories^8^.

**Figure 2:**
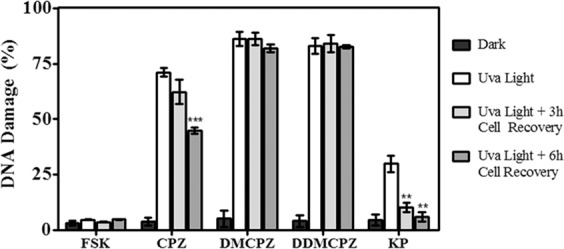
Alkaline single cell gel electrophoresis COMET assay of cells treated (10 µM) with CPZ, DMCPZ or DDMCPZ. Cells without treatment (FSK) or cells treated with S-Ketoprofen (KP) were used as negative and positive control, respectively. Cells were treated, incubated for 30 minutes at 37 °C and then left unexposed (Dark, ◼), irradiated at 2 J/cm^2^ dose (UVA Light, ◻) or irradiated at 2 J/cm^2^ dose followed by 3 h or 6 h of cell recovery: UVA Light + 3 h cell recovery, ◼ and UVA Light + 6 h cell recovery, ◼, respectively. The percentage of DNA damage was obtained for compounds tested by visual scoring. Data are the mean ± SD of 4 independent experiments. Asterisks denote significant differences relative to treated and irradiated FSK cells by means of the t-Student test (**p < 0.01; ***p < 0.001).

As shown in Fig. 2 and Supplementary Fig. S1, DNA damage decreased with the time of recovery only for CPZ whereas the *N*-demethylated metabolites remained unchanged. Interestingly, for the parent drug CPZ it was observed a significant reduction in DNA damage following 6 h recovery. By contrast, for DMCPZ and DDMCPZ no substantial decrease of DNA damage was noticed after 6 h of cell recovery.

**Figure 3:**
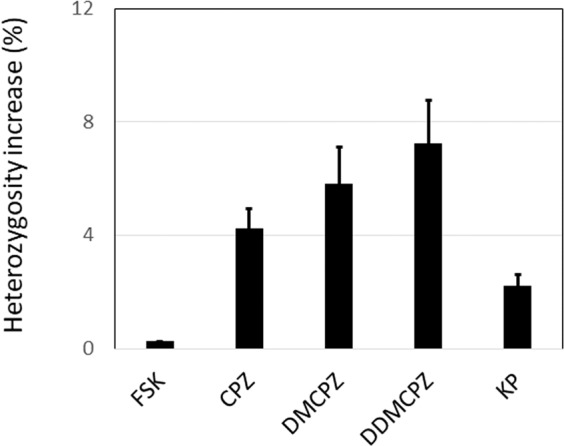
Extent of mutagenesis assessed with NGS. Each bar shows the mean ± SE in the increase of heterogeneity (deviations from 50%) for all SNVs observed in heterozygosity in this study (Table 2). T-Test indicated statistically significant differences (p < 0.001) for all comparisons between irradiated drug-treated cells versus irradiated FSK-cells.

The gene areas sequenced contained several polymorphic regions and all polymorphisms identified were analyzed to assess mutagenicity. The gene locations on Assembly GRCh37 and identification numbers for the gene variations when available are shown in Table 2. Every irradiated sample (T = 5) was compared to a non-irradiated sample with identical composition of chlorpromazine or metabolites (T = 0). Mutagenesis was, therefore, assessed as the net difference between the percentage of the increase in heterogeneity for every variant at time = 5 versus time = 0 for the seven SNVs found in heterozygosity. Then, the average of all net differences in heterozygosity for all the SNVs mentioned above was used to assess the mutagenesis effect as shown in Fig. 3.

**Table 2:** Gene variations analyzed.

Position	Random SNP Identification	Hereozygous in the FSK cell line	Type of variant	Percentage of heterozygosity under basal conditions
chr13:32907420	rs397507608	No	—	—
chr13:32907615	–	Yes	A/ATATCT	49.12 ± 3.36
chr13:32912299	rs543304	Yes	C/T	51.65 ± 3.72
chr13:32912345	rs80359406	No	—	—
chr13:32913055	rs206075	yes	C/T	49.42 ± 1.75
chr13:32915005	rs206076	Yes	C/G	48.55 ± 2.47
chr13:32929387	rs169547	Yes	C/T	48.21 ± 2.66
chr13:32944741	rs11571744	Yes	C/T	48.24 ± 2.94
chr13:32954302	–	No	—	—
chr13:32968808	–	No	—	—
chr17:41244936	rs799917	Yes	T/C	49.37 ± 2.56
chr17:41245586	–	No	—	—
chr17:41276170	–	No	—	—

Chromosomal locations correspond to the Assembly GRCh37. Basal conditions represent non-irradiated and non-drug exposed cells.

Our findings show that the extent of DNA changes as assessed with NGS was similar as that observed by the COMET assay, as shown in Fig. 3: Cells without treatment (FSK) showed negligible DNA changes, the effect of CPZ lower than that of DMCPZ and DDMCPZ and, finally, the effect of KP is low. A minor discrepancy, however, is observed with DDMCPZ, which seems to cause more DNA changes as assessed by NGS than that assessed with the COMET assay. We have no explanation for this difference, although we could speculate that NGS could provide higher sensitivity or a higher discrimination capacity when a high level of DNA damage is achieved. Even with the minor discrepancy observed with DDMCPZ, the Pearson Correlation Coefficient (R value) for the comparison between the results obtained by NGS and the COMET assay is very high = 0.9530. Although additional comparative studies are required to prove whether NGS can be even more sensitive than the COMET assay, our findings confirm that NGS has potential for DNA damage assessment thus adding more uses to this powerful sequencing technique.

It should be stated that, because DNA is collected shortly after cell irradiation, the DNA changes observed by NGS are not fixed in the cell genome and, therefore, the presence of mutations in the NGS study does not mean that these mutations will be permanent. Permanent mutations cannot be detected after cell treatment with drug or its metabolites and irradiation, because this combination promotes cell death in FSK cells after 24 h assessed by trypan-blue exclusion assay (data not shown). Indeed, high cell viability is a requirement to avoid misleading results as cell death also promotes DNA fragmentation by activation of caspase-activated DNases (CADs). We analyzed DNA damage 3 h after cells were incubated with drug or metabolites followed by UVA irradiation, when cell viability is higher than 85%. Therefore, it may be argued that the mutations detected by means of NGS may result from an artifact of amplification of damaged DNA. However, this is unlikely because the total number of reads, bases and the coverage of the NGS experiments are similar in irradiated and non-irradiated cells (Table 1). Therefore, although the DNA changes detected by NGS are not permanent, they reflect DNA variation after irradiation. Another potential limitation is that our findings are based on the seven SNVs observed in heterozygosity in the cells, and that this procedure is limited as compared to other strategies such as classic shuttle vectors, or even specific genes sequenced individually after cloning in bacteria. However, NGS procedures have the advantage of the speed of the process, and the correlation of the results obtained by NGS assessment and the COMET assay is, at least in this pilot study, very high.

In conclusion, the combination of the single cell gel electrophoresis (COMET) assay and next generation sequencing (NGS) has been successfully applied to assess the photomutagenicity of CPZ, a well-known neuroleptic drug, in comparison with its *N*-demethylated metabolites DMCPZ and DDMCPZ. Upon exposure to the UVB-UVA fraction of ultraviolet radiation contained in sunlight, the COMET assay reveals CPZ-photosensitized DNA damage that is partially repaired by cells after several hours. By contrast with this result, the metabolites DMCPZ and DDMCPZ mediate extensive DNA- photodamage, which is hardly repaired under the same conditions. Parallel assessment of the extent of DNA changes by means of NGS are consistent with these observations, showing the same trend with minor discrepancies for DDMCPZ. To our knowledge, this is the first example demonstrating the utility of NGS for assessing drug-induced photomutagenicity. Further NGS studies in different comet DNA fractions separately, might refine the potential of NGS in photomutagenicity assessment.

## Supplementary information

Supplementary Information.
