# Supplementary material for: Photomutagenicity of chlorpromazine and its N-demethylated metabolites assessed by NGS
Source: Sci Rep. 2020 Apr 23;10:6879. doi: 10.1038/s41598-020-63651-y (PMC7181754; doi:10.1038/s41598-020-63651-y)
Supplement: Supplementary file 1 — Supplementary Information. [file 41598_2020_63651_MOESM1_ESM.pdf]

Photomutagenicity of chlorpromazine and its *N*-demethylated metabolites assessed by NGS

José A. G. Agúndez,[1] Elena García-Martín,[1] Guillermo García-Lainez,[2] Miguel A. Miranda[3,4,*] and Inmaculada Andreu[3,4,*]

[1]University Institute of Molecular Pathology Biomarkers, UEx. ARADyAL, Instituto de Salud Carlos III, 10003, Cáceres. Spain

[2]Instituto de Investigación Sanitaria (IIS) La Fe, Hospital Universitari i Politècnic La Fe, Avenida de Fernando Abril Martorell 106, 46026, Valencia, Spain

[3]Departamento de Química-Instituto de Tecnología Química UPV-CSIC. Universitat Politècnica de València, Camino de Vera s/n, Apdo 22012, 46071, Valencia, Spain

[4]Unidad Mixta de Investigación UPV-Instituto de Investigación Sanitaria (IIS) La Fe, Hospital Universitari i Politècnic La Fe, Avenida de Fernando Abril Martorell 106, 46026, Valencia, Spain

*****Correspondence should be addressed to M. A. M. (mmiranda@qim.upv.es) or I. A. (iandreur@qim.upv.es).

-Supplementary Information-

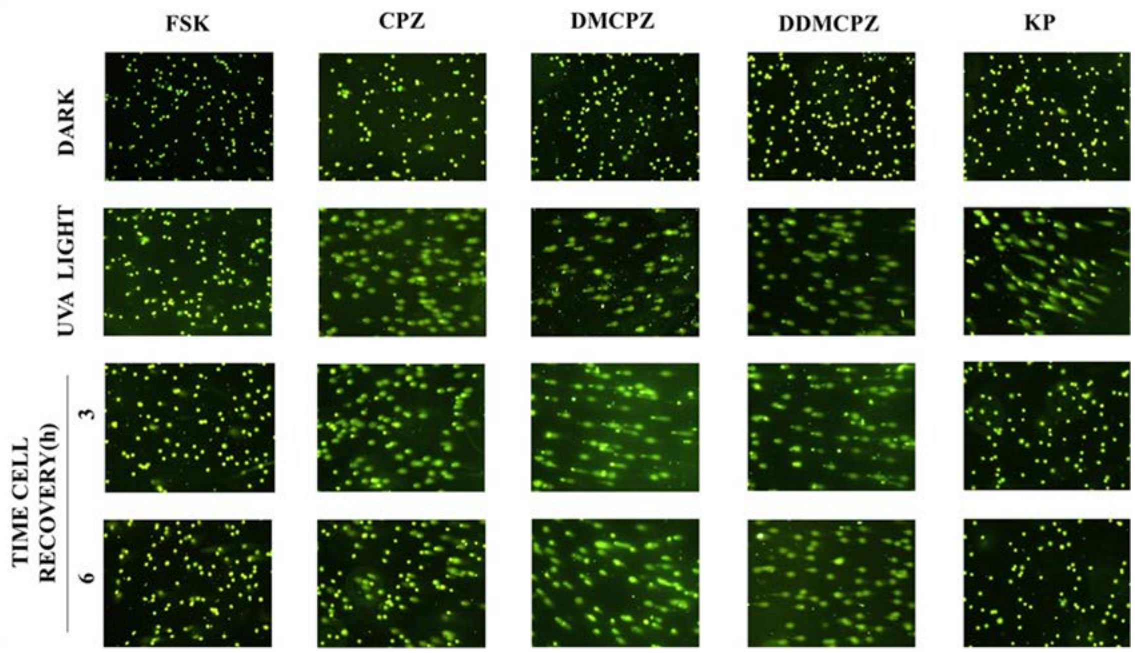

Figure S1. Microscopy images of the comet assay experiments. Dark: Unexposed FSK alone (negative control, NC) or treated with CPZ and its metabolites at 10 µM. UVA Light: Untreated or incubated FSK cells with CPZ and its metabolites after 5 minutes of UVA irradiation (dose: 2J/cm^2).
